# An Immunocompetent HIV-Negative Elderly Patient with Low-Grade Fever, Generalized Lymphadenopathy, Splenomegaly, and Acute Phase Response: Do Not Forget Castleman Disease

**DOI:** 10.1155/2021/6614208

**Published:** 2021-03-11

**Authors:** Kalliopi Azariadis, Maria Ioannou, Kalliopi Zachou, George N. Dalekos

**Affiliations:** ^1^Department of Medicine and Research Laboratory of Internal Medicine, National Expertise Center of Greece in Autoimmune Liver Diseases, General University Hospital of Larissa, 41110 Larissa, Greece; ^2^Department of Pathology, Medical School, University of Thessaly, 41110 Larissa, Greece

## Abstract

Multicentric Castleman disease (MCD) is a rare lymphoproliferative disorder that mainly affects middle-aged patients with human immunodeficiency virus (HIV) infection. However, HIV-negative patients can also be affected representing a small proportion of the total MCD cases. Of note, recent studies from China in HIV-negative patients with MCD have suggested that the onset of the disease can be observed in younger age than previously thought. If undiagnosed and untreated, the MCD has a poor prognosis and may progress to lymphoma. We present an 82-year-old immunocompetent male patient who was admitted to our department because of low-grade fever, cachexia, anasarca, hepatosplenomegaly, and generalized lymphadenopathy. Laboratory findings showed anemia and increased markers of inflammation including hyperferritinemia and polyclonal hyperglobulinemia. Infectious causes including HIV were ruled out. Histological examination of a cervical lymph-node revealed lesions supportive of MCD diagnosis. Of note, the outer-zone plasmablasts' nuclei stained positive for human herpesvirus-8 (HHV8). The patient received 4 cycles of cyclophosphamide, vincristine, and dexamethasone with regression of all symptoms. This case underlines that HHV8-associated MCD should be considered as a rare cause of generalized lymphadenopathy even in HIV-negative immunocompetent patients when other causes have been appropriately excluded because a timely diagnosis can be life-saving.

## 1. Introduction

Castleman disease (CD) was first described in 1954 in a series of patients with localized mediastinal lymphadenopathy and characteristic histopathological features [1]. Currently, the term CD is applied to diverse polyclonal B-cell lymphoproliferative entities with distinct clinicopathologic features based on the number of affected lymph nodes. The disease is classified in two general forms: the unicentric CD (UCD) and the multicentric CD (MCD) [2–5]. The UCD form refers to 75% of all cases, with histological features of either hyaline vascular proliferation (90%) or mature plasma cell proliferation (10%) [6, 7]. The MCD is subclassified to (a) the human herpes virus 8 (HHV8 or Kaposi Sarcoma- (KS-)associated herpes virus, KSV) positive MCD seen predominantly in human immunodeficiency virus- (HIV-) infected or otherwise immunocompromised individuals [2, 4, 5, 8] and (b) the idiopathic MCD (iMCD) with histological features of both hyaline vascular and plasma cell proliferation with relatively preserved nodal architecture [3, 9]. In addition, two clinical syndromes have been described in association with iMCD: POEMS syndrome (polyneuropathy, organomegaly, endocrinopathy, M-proteins, and skin changes) [10, 11] and TAFRO syndrome (thrombocytopenia, anasarca, fever, reticulin fibrosis, and organomegaly) [10, 12].

The estimated annual incidence of MCD ranges between 2.4 and 6.25 per million person-years in Western population [12–14]. However, HHV8-associated MCD prevalence is practically unknown and probably underestimated, being underdiagnosed especially in HIV-negative patients who represent only a small proportion of cases [15–17]. The reasons for underdiagnosis is the high level of suspicion required from clinicians and pathologists, as the clinical features of HHV8-associated MCD overlap significantly with those of uncontrolled infections and lymphoid malignancies. The clinical and laboratory characteristics include systemic manifestations such as fever, sweating, fatigue, cachexia, generalized lymphadenopathy, splenomegaly, anasarka, cytopenias, and hypoalbuminemia [18–21]. Furthermore, patients with MCD are at high risk of developing non-Hodgkin lymphomas (NHL) [11, 21, 22]. Risk assessment and estimation of survival is difficult because of the rarity of the disease. However, a 5-year survival of about 80% has been recorded after treatment in small case series from France and South Korea [11, 23].

Herein, we present a case of an elderly immunocompetent patient with HHV8-associated MCD and highlight the crucial steps for establishing the diagnosis in conjunction with approach to treatment.

## 2. Case Presentation

An 82-year-old male was admitted to the Department of Medicine because of low-grade fever (37.8^o^C) over the past 24 hours and cachexia, occipital headache, and pain in the cervical spine over the past 72 hours. His medical history included ischemic heart failure under furosemide and bisoprolol, atrial fibrillation under dabigatran, ocular cataract, and a single episode of herpes zoster in the left abdominal wall four months ago. The patient resided in an agricultural area, owned poultry, and reported frequent consumption of nonpasteurized dairy products. On clinical examination, the patient was hemodynamically stable, and he had anasarka and tenderness at the cervical spine without stiffness, restriction to movement or Kerning and Brudzinski signs. The rest neurologic examination was also normal. The liver and spleen were palpable in combination with multiple nontender, mobile lymph nodes in the cervical, supraclavicular, axillary, and inguinal areas.

Laboratory work-up on admission revealed hypochromic normocytic anemia, with normal leukocytes and platelets count, hypoalbuminemia, polyclonal hyperglobulinemia with increased immunoglobulin G and increased inflammatory markers such as erythrocyte sedimentation rate, C-reactive protein, ferritin, and fibrinogen (Supplementary Table S1). The rest of the laboratory tests were within normal limits (Supplementary Table S1). The chest X-ray revealed bilateral pleural effusions. Because of the presence of low-grade fever, headache, and tenderness at the cervical spine, a cerebrospinal fluid paracentesis was performed which was not contributory (Supplementary Table S1). To further assess the generalized peripheral lymphadenopathy, a computer tomography (CT) of the cervix, thorax, and abdomen was performed. The results showed several lymph nodes (maximum diameter 3.8 cm) with non-necrotizing centers in all anatomical spaces (cervix, supraclavicular and axillary areas, mediastinum, and retroperitoneal, femoral, and inguinal areas). A work-up for zoonosis based on local endemicity [24–29] that included serological tests for *Brucella*, *Leishmania*, *Leptospira*, *Coxiella species*, *Rickettsia conorii* and *typhi*, *Bartonella*, and *Toxoplasma* was unrevealing (Supplementary Table S1). Furthermore, serological testing for several viruses including Epstein Barr Virus, cytomegalovirus, HIV, and hepatitis B and C viruses was also negative. Multiple sets of blood cultures incubated for 21 days for slowly growing bacteria were also negative. Tuberculosis was ruled out based on a negative skin tuberculin test and negative sputum examination (direct Ziehl-Neelsen staining and long-term culture of 6 weeks). Peripheral blood and bone marrow smears were negative for neoplastic cells while the bone marrow biopsy revealed granulocyte left shift, small T and B cells, and 8% polyclonal plasma cells.

Finally, a right supraclavicular lymph node of 3.8 cm diameter was biopsied. The histological examination revealed mildly deranged nodal architecture, multiple lymphoid follicles with vascular proliferation and perivascular hyalinization of the blast centers, increased follicular dendritic cells (CD21+), and mantle lymphocytes in onion-skin layers surrounded by polyclonal plasma cells without monoclonal proliferation of *κ*- and *λ*-light chains. Staining for CD3, CD4, CD68, ALK, EMA, CD23, and cyclin D1 was nonsupportive of lymphoma. Of note, anosoblasts/plasmablasts (MUM1+, CD30+, and CD15-) in the outer zone showed positive nuclear staining for HHV8.

**Figure 1:**
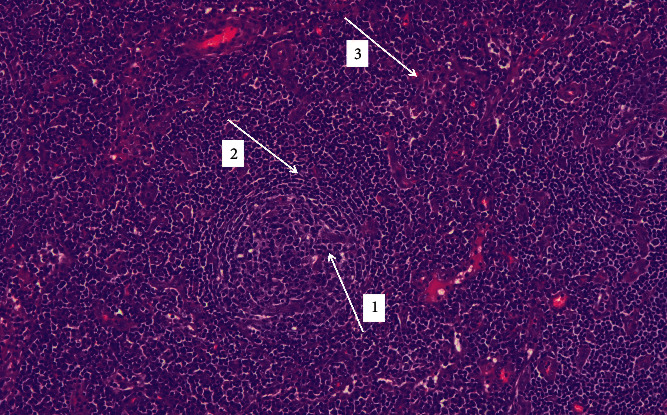
Hematoxylin and eosin stain, original magnification ×10. Germinal centers traversed by penetrating vessels (arrow 1) and thickened mantle zones with lymphocytes arranged in layers with onion skin appearance (arrow 2). In the interfollicular areas, there is extensive vascular proliferation with perivascular hyalinization (arrow 3).

**Figure 2:**
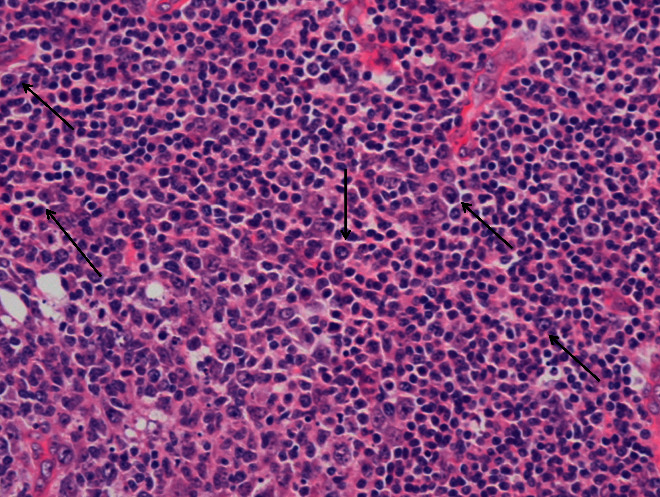
Hematoxylin and eosin stain, original magnification ×40. Sheets of mature plasma cells and a few plasmablasts with large nuclei, vesicular chromatin, and prominent nucleoli (arrows) are also seen in the interfollicular areas.

**Figure 3:**
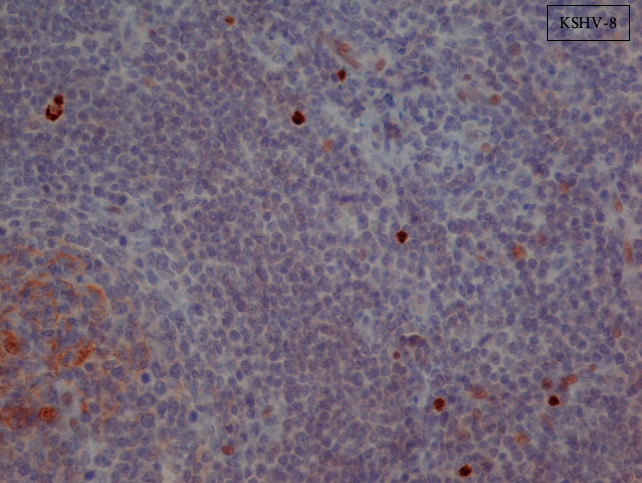
HHV-8 immunohistochemical stain, original magnification ×40. Cells with plasmablastic morphology are HHV8-infected cells (brown nuclei). These cells were polytypic (immunostaining for kappa and lambda light chains (data not shown)). In order to exclude the possibility of random positive counting, the immunostaining section was split into four fields and cells positive for HHV8 were counted in each of these four quartiles. The measurements were grouped for each quartile. Pairwise comparisons among groups using the student *t* test showed *p* values >0.05 in all cases. In addition, repeating procedures concerning immunohistochemical staining for HHV8 were performed in other two different histological sections producing finally similar results.

As most of the causes of generalized lymphadenopathy in combination with hepatosplenomegaly, anasarka, high markers of acute phase response, and polyclonal hyperglobulinemiahad been excluded, the differential diagnosis included sarcoidosis and miscellaneous lymphoproliferative disorders. However, sarcoidosis was unlikely based on the lack of abnormal findings in the lung imaging, absence of respiratory symptoms, and nontypical lymph node lesions on histology. In addition, no arthritis was mentioned or was evident on physical examination. In contrast, the histological findings of the lymph node biopsy revealed a benign lymphoproliferative disorder characterized by increased plasma cells that stained positive for HHV8 along with vascular proliferation and perivascular hyalinization supporting the diagnosis of CD (Figures 1–3).

The patient received four cycles of cyclophosphamide (1000 mg/cycle), vincristine (10 mg/cycle), and dexamethasone (16 mg/day for days 1–5/cycle) in 21-day intervals. Resolution of symptoms and all physical findings was achieved after the third cycle. Normalization of the acute phase inflammatory markers and resolution of anemia was evident at the end of the fourth cycle indicating complete response at 12 weeks of treatment (Supplementary Table S1). Complete remission of the disease was further confirmed on 18F-FDG positron emission tomography. Regarding side effects, the patient experienced only a single episode of neutropenia after the second cycle of treatment which required administration of granulocyte colony-stimulating factor (GCSF). Till the time of this writing, the patient remains asymptomatic having completed 6 months of follow-up without any clinical or laboratory sign of recurrence.

## 3. Discussion

HHV8-associated MCD is a rare clinical entity, especially in HIV-negative immunocompetent individuals. The incidence of HHV8-associated MCD is highly related to the HHV8 endemicity in the general population (1.4–1.9% in Japan, 4% in north Europe, and 10–20% in the Mediterranean basin and Balkans) [30–36]. Furthermore, the demographic characteristics of HIV-negative, HHV8-assosciated MCD patients differ according to the population studied with European and American studies reporting higher incidence in males in the sixth decade [10, 12, 23, 31]. However, a recent retrospective study from China in 185 CD patients including 64 with MCD has shown that the onset of the disease can be observed in younger age, even though precise demographic data on the 11/64 HIV-negative, HHV8-associated MCD patients was not given [32].

The clinical characteristics of the disease include the presence of generalized lymphadenopathy, splenomegaly, and systemic inflammatory manifestations such as fever, fatigue, night sweats, weight loss, volume overload (sometimes with ascites and pulmonary effusions), skin abnormalities (including hyperpigmentation and cherry hemangiomas), and nonspecific neurologic, respiratory, and gastrointestinal symptoms. Secondary hemophagocytic lymphohistiocytosis (sHLH), autoimmune cytopenias, renal involvement (including secondary amyloidosis and membranoproliferative glomerulonephritis), peripheral neuropathy, lymphoid interstitial pneumonitis, and bronchiolitis obliterans have also been described [33, 35, 37]. Of note, patients with HHV8-associated MCD are at high risk of developing HHV8-associated lymphomas and KS even years after the initial diagnosis [22, 38].

Regarding MCD pathogenesis, overproduction and dysregulation of circulating cytokines like interleukin-6 (IL-6), interleukin-10, tumor necrosis factor-alpha, and interleukin-1 are thought to play a pivotal role, with IL-6 being the key playmaker [2, 39]. In addition to its apparent role as a growth and differentiation factor for lymphocytes and plasma cells leading to lymph node enlargement, hepatosplenomegaly, and bone marrow polyclonal plasmacytosis with the accompanying B-symptoms (fever, night sweats, weight loss, and cachexia), IL-6 exhibits many pleiotropic actions [40]. Hepatocytes respond to IL-6 by diminishing albumin production and producing several acute phase proteins and hepcidin, which in turn leads to reduced intestinal iron absorption, impaired release of iron stored in the macrophages, and anemia [40, 41]. In addition, the endothelium responds by increasing vascular endothelial growth factor secretion which induces angiogenesis and increased vascular permeability. The latter, in combination with the hypoalbuminemia, precipitates extravascular fluid accumulation (ascites, pleural and pericardial effusion, and anasarka). Furthermore, increased fibrinogen and tissue factor production can cause hypercoagulopathy, thromboembolic phenomena, and thrombotic microangiopathy leading to multiorgan failure [40].

HIV infection or other immunosuppressive state enables HHV8 to escape the host's immune system and replicate in lymph node plasmablasts. Lytic activation of infected B-cells results in release of cytokines which in turn gives rise to the clinical syndrome [2]. Interestingly, HHV8-associated MCD can also occur in HIV-infected patients with preserved CD4 counts and low HIV-RNA [42]. In immunocompetent individuals, the cause that drives HHV8 escape is largely unknown, as it is not clear why individuals infected with HHV8 only rarely develop MCD [2]. In more detail, HHV8 has tropism for B cells, monocytes, dendritic cells, keratinocytes, and epithelial and endothelial cells [8]. Several HHV8 viral proteins enhance the production of human IL-6 (hIL-6) or independently activate hIL-6 pathways [2, 8]. HHV8-encoded viral IL-6 (vIL-6), which is mainly released during the lytic replication of the virus, bears a 25% sequence homology with hIL-6 and can bind directly to the IL-6 receptor (gp130) irrespectively of its co-receptor p80 [8, 43]. Therefore, it is possible that vIL6 enhances IL-6 signaling in a wider range of tissues compared to hIL-6. In HHV8-associated MCD, vIL-6 plasma levels correlate with disease activity and collaboration of both viral and endogenous IL-6 may be crucial for the clinical expression of MCD [2, 8].

A thorough investigation and exclusion of several infectious diseases based on local epidemiology, autoimmune diseases, sHLH [44], and hematological neoplasms, as we performed in our patient, is essential for the differential diagnosis of the disease. HIV serology is mandatory in all patients, since management and prognosis differ in the case of HIV-positive HHV8-associated MCD. Finally, a lymph node biopsy is mandatory in order to exclude NHL and guide the diagnosis and treatment. Positive immunohistochemical staining for HHV8 and/or PCR for HHV8 establishes the diagnosis of HHV8-associated MCD in a patient with generalized lymphadenopathy and CD histopathology.

If untreated, HHV8-associated MCD bares a dismal prognosis and can be lethal within two years [44]. However, due to the rarity of the disease, there is no established treatment [5, 45]. For this reason, the outstanding Castleman Disease Collaborative Network (CDCN) was founded in 2012 by Drs Frits van Rhee and David Fajgenbaum, in order to advance research on pathophysiology, diagnosis, and especially treatment. In the premonoclonal antibody era, therapy was based on cytotoxic chemotherapy, extrapolated from the treatment of B-cell lymphomas. Agents used in different anecdotal cases and small series include etoposide, interferon-alpha, thalidomide, cyclophosphamide, vincristine, and doxorubicin with or without corticosteroids in various combinations [37, 38, 45]. Conclusions regarding efficacy, response, and relapse rates cannot be drawn safely due to the lack of randomized trials, established response criteria, and the heterogeneity of patients. The role of antivirals (ganciclovir, foscarnet, cidofovir, and valanciclovir) is controversial, and remissions have been described only with the use of ganciclovir in one small retrospective study [45, 46].

Breakthrough in MCD treatment was the discovery of the biological agents. Several studies in HIV-positive HHV8-associated MCD patients support the use of rituximab, whereas efficacy in HIV-negative patients relies mostly on anecdotal cases or small case series [4, 5, 47–50]. Treatment with rituximab has also been associated with a substantially lower risk of progression to NHL, although bearing the risk for KS exacerbation in patients with concurrent KS [5, 45]. Biological agents targeting the pathogenetic IL-6 pathway have shown efficacy in iMCD, but the clinical experience with these agents in HHV8-associated MCD is scarce [45]. Actually, tocilizumab, a humanized monoclonal antibody against the IL-6 receptor has shown efficacy and long-term safety in two HIV-negative HHV8-associated MCD cases [51]. As of September 2011, an open-label clinical trial to assess efficacy of tocilizumab in HHV8-associated MCD (NCT01441063) has been running in the USA, with 8 patients enrolled and estimated completion date in 2023.

Nevertheless, the treatment of HHV8-associated MCD should be individualized keeping in mind the lack of consensus on response-to-treatment criteria and optimal treatment duration [5, 45]. Our patient was symptomatic with increased inflammatory markers, and he received four cycles of cyclophosphamide, vincristine, and dexamethasone. We have chosen this treatment option because of the potential high risk of rituximab toxicity in a patient older than 80 years of age keeping also in mind, that in the case series of rituximab administration in patients with HHV8-associated MCD, the patients were much younger than our patient. The response was spectacular, since all symptoms and signs resolved and laboratory values returned to normal after four months of treatment. In addition, the patient experienced only a single episode of neutropenia after the third circle that required treatment with GCSF. Complete treatment response was confirmed by 18F-FDG PET, and the patient remains on remission six months after treatment completion.

In conclusion, our case highlights the importance for clinicians to keep in mind and recognizes this rare syndrome even in immunocompetent patients with generalized lymphadenopathy and systemic symptoms when other common causes have been appropriately excluded because a prompt and timely diagnosis can be life-saving as in our case. Lymph node histology is the backbone for a firm diagnosis and exclusion of other pathologies. HHV8-associated MCD should not be overlooked as this disorder carries a malignant course and unfavorable prognosis if undiagnosed and untreated.

## Data Availability

The data used to support the findings of this study are included within the article.

## Supplementary Materials

Supplementary Materials Table S1: laboratory data of the patient on admission and after remission (12 weeks after treatment initiation).
