# Supplementary material for: An Immunocompetent HIV-Negative Elderly Patient with Low-Grade Fever, Generalized Lymphadenopathy, Splenomegaly, and Acute Phase Response: Do Not Forget Castleman Disease
Source: Case Rep Infect Dis. 2021 Mar 11;2021:6614208. doi: 10.1155/2021/6614208 (PMC7979292; doi:10.1155/2021/6614208)
Supplement: Supplementary Materials — Table S1: laboratory data of the patient on admission and after remission (12 weeks after treatment initiation). [file 6614208.f1.docx]

**Supplementary Table S1.** Laboratory data of the patient on admission and after remission (12 weeks apart treatment initiation)

|  | | *On admission* | *12 weeks after treatment initiation* | *Reference values* |
| --- | --- | --- | --- | --- |
| White cell count (x10^3^/uL) | | 6.1 | 6.85 | 4.5-10.5 |
| Neutrophils (x10^3^/uL)  Lymphocytes (x10^3^/uL)  Monocytes (x10^3^/uL)  Eosinophils (10^3^/uL)  Basophils (x10^3^/uL) | | 3.66  1.22  0.9  0.05  0.01 | 2.75  2.7  0.93  0.27  0.07 | 1.5-6.5  1.2-3.8  0.2-1  0-0.7  0-0.2 |
| Red cell count (x10^6^/uL) | | 3.02 | 4.19 | 3.8-5.4 |
| Hematocrit (%) | | 29.7 | 40.2 | 39-46 |
| Hemoglobin (g/dL) | | 9.8 | 13.2 | 13-16 |
| Mean corpuscular volume (fl)  Mean corpuscular hemoglobin (pg)  Mean corpuscular hemoglobin concentration (g/dL) | | 98  32  32 | 96  31.6  32.9 | 79-98  26-32  32-36 |
| Platelets (x10^3^/uL) | | 196 | 211 | 140-440 |
| Erythrocyte sedimentation rate (mm/1h) | | 40 | 10 | 2-15 |
| Prothrombin time (sec)  International normalized ratio | | 23*  2.05* | 18*  1.8* | 9-14  0.85-1.15 |
| Activated partial thromboplastin time (sec) | | 56 | 40 | 25-35 |
| D-dimers (ng/mL) | | 216 | 116 | <225 |
| Fibrinogen (mg/dL) | | 510 | 200 | 180-440 |
| C-reactive protein (mg/dL) | | 7.2 | 0.03 | 0-0.7 |
| Ferritin (ng/mL) | | 822 | 300 | 20-300 |
| Urea (mg/dL) | | 43 | 61 | <43 |
| Creatinine (mg/dL) | | 1.2 | 1.19 | 0.7-1.2 |
| Glucose (mg/dL) | | 129 | 90 | 75-100 |
| Sodium (mmol/L) | | 136 | 139 | 136-146 |
| Potassium (mmol/L) | | 5 | 4.38 | 3.5-5.3 |
| Calcium (mg/dL) | | 8.8 | 9.1 | 8.8-10.4 |
| Total protein (g/dL) | | 6.55 | 6.96 | 6.4-8.3 |
| Albumin (g/dL) | | 3.3 | 3.98 | 3.5-5.2 |
| Bilirubin (mg/dL) | | 0.7 | 0.8 | <1.1 |
| Aspartate aminotransferase (IU/L) | | 20 | 16 | <40 |
| Alanine aminotransferase (IU/L) | | 16 | 8 | <40 |
| Alkaline phosphatase (IU/L) | | 59 | 53 | <120 |
| Gamma-Glutamyl Transferase (IU/L) | | 12 | 16 | <38 |
| Creatine phosphokinase (IU/L) | | 20 | 75 | <145 |
| Lactate dehydrogenase (IU/L) | | 160 | 184 | <247 |
| Uric acid (mg/dL) | | 4.3 | 5.5 | 3.5-7.2 |
| Cholesterol (mg/dL)  HDL cholesterol (mg/dL)  Triglycerides (mg/dL) | | 66  8  199 | 100  10  242 | <200  >45  <150 |
| IgG (mg/dL)  IgA (mg/dL)  IgM (mg/dL) | | 1740  218  76 | 1530  213  65 | 847-1650  99-300  64-249 |
| Thyroid stimulating hormone (uIU/mL)  Free thyroxine (ng/dL) | | 2.7  1.8 |  | 0.27-4.2  0.7-2 |
| C3 complement component (mg/dL)  C4 complement component (mg/dL) | | 100  35 |  | 90-180  10-40 |
| Anti-nuclear antibodies | | negative |  | <1/80 |
|  | ***Serological tests for infections*** | | | |
| HIV 1/2 antibodies | | negative |  | negative |
| Wright-Coombs | | negative |  | negative |
| Leishmania (PCR) | | negative |  | negative |
| Leptospira antibodies IgG/IgM | | negative/negative |  | negative |
| Coxiella species antibodies IgG/IgM | | negative/negative |  | negative |
| Rickettsia conorii antibodies IgG/IgM Rickettsia typhi antibodies IgG/IgM | | negative/negative  negative/negative |  | negative  negative |
| Bartonella antibodies IgG/IgM | | negative/negative |  | negative |
| Toxoplasma antibodies IgG/IgM | | negative/negative |  | negative |
| Epstein Bar virus antibodies IgG/IgM | | negative/negative |  | negative |
| Cytomegalovirus antibodies IgG/IgM | | negative/negative |  | negative |
| Hepatitis B surface antigen  Hepatitis B surface antibodies (mIU/mL)  Hepatitis B core antibodies IgG | | negative  0  negative |  | negative  >10  negative |
| Hepatitis C virus antibodies | | negative |  | negative |
|  | ***Cerebrospinal fluid analysis*** | | | |
| Nucleated cells (/mm^3^) | | 2 |  | <5 |
| Red blood cells (/mm^3^) | | 0 |  | <5 |
| Protein (mg/dL) | | 32 |  | 15-45 |
| Glucose (mg/dL) | | 70 |  | 50-75 |

*the patient was receiving dabigatran for atrial fibrillation. PCR, polymerase chain reaction.
